# A review of deep learning approaches for multimodal image segmentation of liver cancer

**DOI:** 10.1002/acm2.14540

**Published:** 2024-10-07

**Authors:** Chaopeng Wu, Qiyao Chen, Haoyu Wang, Yu Guan, Zhangyang Mian, Cong Huang, Changli Ruan, Qibin Song, Hao Jiang, Jinghui Pan, Xiangpan Li

**Affiliations:** ^1^ Department of Radiation Oncology Renmin Hospital Wuhan University Wuhan Hubei China; ^2^ School of Electronic Information Wuhan University Wuhan Hubei China

**Keywords:** deep learning, liver cancer, multimodal fusion technology, segmentation

## Abstract

This review examines the recent developments in deep learning (DL) techniques applied to multimodal fusion image segmentation for liver cancer. Hepatocellular carcinoma is a highly dangerous malignant tumor that requires accurate image segmentation for effective treatment and disease monitoring. Multimodal image fusion has the potential to offer more comprehensive information and more precise segmentation, and DL techniques have achieved remarkable progress in this domain. This paper starts with an introduction to liver cancer, then explains the preprocessing and fusion methods for multimodal images, then explores the application of DL methods in this area. Various DL architectures such as convolutional neural networks (CNN) and U‐Net are discussed and their benefits in multimodal image fusion segmentation. Furthermore, various evaluation metrics and datasets currently used to measure the performance of segmentation models are reviewed. While reviewing the progress, the challenges of current research, such as data imbalance, model generalization, and model interpretability, are emphasized and future research directions are suggested. The application of DL in multimodal image segmentation for liver cancer is transforming the field of medical imaging and is expected to further enhance the accuracy and efficiency of clinical decision making. This review provides useful insights and guidance for medical practitioners.

## 1 INTRODUCTION

### 1.1 Epidemiology and clinical features of liver cancer

The liver is one of the most important organs for metabolism in the body, with vital functions such as vitamin metabolism, hormone metabolism, detoxification, anticoagulation and coagulation, and bile secretion and excretion. It also has an immunoprotective role for the body due to the large amount of blood sinusoids that contain many Kupffer cells.^1^, ^2^

However, the liver's rich functionality also brings a high chance of tumor formation. A study by Villanueva showed^3^ that globally, liver cancer is the fourth leading cause of death from cancer and the sixth most common cancer; based on annual predictions, the World Health Organization estimates that more than 1 million patients will die of liver cancer by 2030; in the United States, between 2000 and 2016, the death rate from liver cancer increased by 43% (from 7.2 to 10.3 deaths per 100,000 people), while the 5‐year survival rate for liver cancer is 18%, making it the second most lethal tumor after pancreatic cancer. In China, liver cancer mortality is the third highest cause of death from malignant tumors, as reported by a survey of malignant tumor mortality trends conducted in the Haidian District of Beijing,^4^ China, in 2021, which is consistent with data from the Global Cancer Observatory 2020 (Globocan2020).

Liver tumors are common malignant tumors, which can be generally divided into primary and secondary liver cancers, and the main pathological types of the former include hepatocellular carcinoma (HCC), intrahepatic cholangiocarcinoma (ICC), and combined hepatocellular‐cholangiocarcinoma (cHCC‐CCA), among which HCC accounts for 75%–85% and ICC accounts for 10%–15%. The latter is usually spread from other organs and is often an advanced tumor.^3^, ^4^, ^5^ HCC is more hidden, and some literature has suggested that HCC may be a sign of clinically hidden asymptomatic liver disease,^6^ so it often lacks typical clinical signs in the early stage. However, if it progresses to the middle or late stage, patients often display systemic and gastrointestinal symptoms such as weight loss, fatigue, liver pain, jaundice, abdominal swelling, and so forth, and even paraneoplastic syndrome (PNS) such as high cholesterol, low blood sugar, and high platelets may occur.^7^ In addition, HCC may also be a sign of hidden and asymptomatic liver disease.

Thus, the data indicate that the impact of liver cancer on global human health cannot be ignored, and the threats of liver tumors to human life and health need to be urgently addressed.

### 1.2 Liver cancer diagnosis and treatment

Medical imaging technology has advanced greatly, enabling accurate examination and diagnosis of liver tumors, which are no longer hindered by their long latency period or the doctors' experience level. The main clinical imaging techniques used today include B‐scan ultrasonography, computed tomography (CT), magnetic resonance (MR), and positron emission tomography (PET) with 18F‐FDG. Dynamic enhanced CT of the abdominal region and multiparametric MRI are especially important for diagnosing and planning the treatment of liver tumors.^8^

There are many different treatments for HCC. Surgical resection, percutaneous transluminal ablation, and liver transplantation are effective and curative methods for early‐stage HCC patients, but most HCC patients do not have obvious symptoms until their tumors reach the late stage, which is usually incurable. Therefore, most patients are not suitable for surgical treatment. For these patients with intermediate to advanced HCC, non‐surgical treatment options such as transcatheter arterial chemoembolization (TACE), radiotherapy, and molecularly targeted drugs are more important.^9^, ^10^, ^11^, ^12^ Among them, radiotherapy has improved a lot in the past few decades, because of the progress in imaging and radiotherapy technologies, such as intensity‐modulated radiotherapy (IMRT), image‐guided radiotherapy (IGRT), and stereotactic body radiotherapy (SBRT). These technologies have changed radiotherapy from rough whole‐liver irradiation with low dose to conformal treatment based on imaging and tumor target delineation. This has increased the effectiveness and reduced the risk of complications, making radiotherapy an essential part of the comprehensive treatment plan for liver tumors.^13^, ^14^ Therefore, a conformal radiotherapy plan for liver tumors based on imaging (“3D‐CRT”) requires medical staff to have the ability to accurately outline the target, design the dose, and range of radiotherapy.

Imaging techniques can detect changes in tissue and organ anatomy and density caused by tumor lesions for early screening and diagnosis, for example, HCC in cirrhotic patients can be diagnosed by imaging due to vascular displacement during the malignant transformation of liver cells, in which non‐malignant lesions (e.g., regenerative and dysplastic nodules) receive blood from the portal system branches while malignant nodules receive blood from the hepatic artery. This allows different CT/MRI enhancement protocols to be designed based on this feature.^15^ However, HCC lesions often have indistinct boundaries and variable shapes, and although they have abundant blood supply, their density may be uneven due to bleeding and necrosis and other changes within the lesion; at the same time, the liver is also hard to identify because it is compressed by many abdominal organs and has small density differences; different contrast agents and scanning equipment may also affect the identification of the liver when imaging. The use of different contrast agents and scanning equipment during imaging can also cause density differences after imaging.^16^, ^17^, ^18^, ^19^ Therefore, image‐based segmentation of the liver and liver tumors is very important because, besides surgical planning and prognosis assessment of the liver, accurate measurement of the liver and liver tumor volume is essential for adjusting the radiation dose and the radiotherapy plan rationally in selective radiotherapy.

### 1.3 The connection between target outlining and artificial intelligence (AI) in liver cancer

In current clinical practice, conventional liver tumor outlining is usually done manually by physicians in computer‐aided diagnostic (CAD) systems (e.g., MIM/ Monaco), which involves manual annotation and segmentation of many CT images (e.g., 4D‐CT), and often needs contrast combined with the results of the MR scanning of the CT images as well, because MR has better resolution for soft tissues than CT. The MR results are often compared with the CT results to outline the CT images. This method takes up a lot of human and material resources, and also requires very high professionalism from doctors, who need to master rich clinical and medical image recognition theory and experience, and avoid personal subjective judgment, to ensure the accuracy and consistency of the outlining results. Therefore, it is valuable to research how to use computer technology to achieve multimodal image fusion (e.g., MRI to CT alignment fusion) and automated tumor segmentation of images.

Therefore, clinical researchers have brought AI technology into the field of medical image processing. AI technology can combine clinical information, image data, and pathology results in a multidimensional way, and improve the effectiveness and accuracy of the use and analysis of medical data, including machine learning (ML) and deep learning (DL) technology, and so forth.^20^

Compared with ML algorithms that require manual extraction of features, DL technology has drawn much attention because of its ability to learn complex representations.^21^Its representative algorithms include convolution neural network (CNN), recursive neural network (RNN), and deep belief network (DBN), and so forth. Among them, CNNs are widely used in the field of image processing due to their multi‐layer structure, which can automatically learn multiple levels of features. Typically, a CNN consists of an input layer, hidden layers, and an output layer. The hidden layers include convolutional layers, pooling layers, and fully connected layers. Convolutional and pooling layers extract high‐dimensional features from feature maps, while the fully connected layer integrates and connects these features. The convolutional layer serves as the core of the algorithm, because the application of them allows the algorithm to use all the information of the feature map itself directly.

Researchers have developed several classic CNN models, including LeNet‐5, AlexNet, VGG‐16, ResNet, and GoogLeNet, all based on the fundamental CNN concept.^22^ Subsequently, more advanced models emerged, such as fully convolutional networks (FCNs), U‐Net, and Deeplab, significantly enhancing DL applications in medical image segmentation. In summary, FCNs excel in semantic segmentation tasks due to their ability to handle arbitrary input sizes and generate pixel‐wise predictions. Unlike traditional models, FCNs replace fully connected layers with convolutional layers, resulting in fewer parameters. However, this efficiency comes at the cost of reduced accuracy during inference. U‐Net, on the other hand, stands out for its low annotation data requirements, high accuracy, and innovative architecture. Nevertheless, it faces computational overhead and space complexity limitations. Additionally, the 2D version processes individual image planes, while the 3D version handles three‐dimensional volume data. Deeplab, a semantic segmentation architecture, builds upon its predecessors. It offers advantages such as high inference speed, improved accuracy, architectural simplicity, and adaptability to custom tasks. Key components include a ResNet backbone, atrous convolutions for dense feature maps, and atrous spatial pyramid pooling (ASPP) for multi‐scale context. Notably, DeepLabv3+ further enhances performance.

Researchers typically classify models on their convolutional dimension, namely, 2D, 2.5D, and 3D. Given that the liver and tumor region is a three‐dimensional structure, relying solely on a 2D network would extract spatial features from individual slices but miss critical information between slices. This approach does not align with the precision medicine goal. Consequently, the development of 2.5D and 3D network applications is crucial for liver tumor research. For instance, DL‐MBIR (deep learning model‐based iterative reconstruction) is a rapid reconstruction algorithm used to approximate the image quality achieved by model‐based iterative reconstruction (MBIR). DL‐MBIR employs a 16‐layer residual CNN, implemented on multiple GPUs and trained using Google TensorFlow. It generates 3D reconstruction results that closely approximate real MBIR images. Additionally, the study proposes 2D, 2.5D, and 3D variants of the DL‐MBIR method. Remarkably, the 2.5D method achieves similar quality to the fully 3D approach while reducing computational costs.^23^ Furthermore, Sabir and his team developed a hybrid ResUNet model, combining ResNet, and UNet architectures within the Monai and PyTorch frameworks. Their innovative enhancements optimize performance, particularly for liver tumor segmentation tasks. By streamlining Monai's functionality, they simplify implementation, enabling faster and more efficient model development. This approach achieves a dice coefficient (DC) value of 0.98% for liver tumor detection and 0.87% for segmentation. Ultimately, it may contribute to early cancer diagnosis and improved patient outcomes.^24^

Overall, advancements in medical image analysis have been significantly influenced by the development of various DL models, including CNN, FCN, DeepLab, U‐net, Transformer, GAN, and SAM. This paper provides a concise summary of the progress made in applying DL to multimodal fusion image segmentation for liver cancer. Its aim is to serve as a valuable reference for future research.

## 2 IMAGE PRE‐PROCESSING AND FUSION METHODS

Multimodal medical image fusion (MMIF) combines images from different sources, such as X‐rays, CT, single‐photon emission computed tomography (SPECT), ultrasound (US), magnetic resonance imaging (MRI), PET, and so on, into one image, where each source has important information.^25^ To understand the biological functions, structures, and processes of tumors better, it is more common to use both functional and structural data from medical images together to produce more useful information that helps in diagnosing and treating diseases. In this section, we will present image preprocessing methods and fusion methods.

### 2.1 Image preprocessing methods

Preprocessing is an essential step for achieving high accuracy with DL models. Images from the two main modalities of 3D image acquisition, CT and MRI, may have artifacts from the patient and the device. So, these artifacts need to be removed before segmenting the liver and liver tumors. We discuss some basic preprocessing steps for MRI and CT images and show their importance as a basic part of liver and tumor segmentation.

#### 2.1.1 Hounsfield unit (HU)

The HU is a unit of measure that indicates the density of tissue in an X‐ray tomography (CT scan) image. To see the region of interest (ROI) in a CT image clearly, it is necessary to limit these pixels to a specific range. Patro^26^ proposed some HU value ranges suitable for liver tumor segmentation, namely, [−100, 400]. Kim,^27^ however, determined that the optimal HU range for automatic liver segmentation using the U‐Net model is [−150, 250].

#### 2.1.2 Histogram equalization

Histogram equalization is a technique for image preprocessing that improves the contrast of digital images. It makes the image look better and more recognizable by changing the distribution of pixel intensity in the image so that the pixel values are spread evenly over the whole gray scale range. Contrast‐constrained adaptive histogram equalization (CLAHE) is one of the most popular HE techniques for medical image enhancement.^28^ The basic idea is to split the image into many small regions, and then do local histogram equalization for each region, thus avoiding noise amplification over the entire image. Also, CLAHE introduces a contrast limit to prevent noise from being over‐enhanced.

Stimper^28^ developed a CLAHE‐based image enhancement technique that can be used for multidimensional images. To get more details from CT images, Yuan^29^ and Anwar^30^ proposed an automatic liver tumor segmentation algorithm based on histogram equalization. Given the similarity between the liver and its neighboring organs, as well as the low contrast of liver textures (including tumors and veins), Zhang proposed a cascaded structure for automatic liver segmentation in CT scans. This enhanced FCN integrates multiple post‐processing methods. The study compared and analyzed the performance of various classical segmentation models as post‐processing steps to improve liver segmentation, including graph‐cut‐based approaches, level‐set‐based methods, and conditional random fields (CRF).^31^

#### 2.1.3 Data normalization

Data normalization (Normalization) is a typical data preprocessing technique for scaling data with different features to a consistent range for improved ML and data analysis. In the training process of DL models, data normalization is applied to bring all features of the data to the same scale, which has equal importance in the training process of the model.^32^ It also helps to reach the optimal point faster and reduce the time needed to train the network.

Batch normalization (BN)^33^ stands as the quintessential normalization technique, normalizing feature maps by computing the mean and variance of batches, heights, and widths to expedite model convergence and mitigate overfitting. Building on BN, numerous other normalization techniques have been developed, including instance normalization (IN)^34^ in RNNs, which normalizes feature maps along channels, widths, and heights; layer normalization (LN)^35^ parameterizes weight vectors for supervised image recognition, generative modeling, and deep reinforcement learning; group normalization (GN)^36^ segments the channels of feature maps into groups, then normalizes them based on the channels, heights, and widths within each group; positional normalization (PN)^37^ derives statistical features along the channel dimension and is frequently employed in generative networks.

Additionally, there are other normalization methods include Min‐Max Scaling, standardization, Z‐score normalization, and so forth.

Min‐Max Scaling, also known as linear normalization, is a commonly used data preprocessing method used to map data to a specific range (usually [0,1] or [−1,1]). This method reduces differences between data by calculating the minimum and maximum values of the data and linearly mapping the data to a specified range. Specifically, assuming you have a single Gaussian variable X with a value range of [$X_{\min},X_{\max}$], you can use the min max scaler to scale it to a new range [a, b]. The Min Max Scaling conversion function for the scaled variable $X^{\prime }$ is as follows

$$X^{\prime }=\left(X-X_{\min}\right)*\frac{b-a}{X_{\max}-X_{\min}}+a$$

Among them, $X^{\prime }$ is the scaled variable, and a and b are the specified minimum and maximum values, so the data will be scaled to a specific range.

Z‐score normalization uses statistical concepts such as mean and standard deviation to produce normalized values from unstructured data.^26^

$$Z=\frac{x_{i}-1}{\sigma }$$

In z‐score normalization, the normalized value Z is calculated by subtracting the mean μ of the data from the original value Xi and dividing by the standard deviation σ. Ahmad^38^ and Rezaei^39^ recommended using z‐score normalization as a preprocessing step to make liver and liver tumor segmentation consistent across different researchers.

#### 2.1.4 Image denoising

Image denoising is an image processing technique that tries to restore the quality and sharpness of an original image from an image that is corrupted by noise. Noise is a random interference caused by different factors during image capture, transfer, or storage, which reduces image quality and impacts later image analysis, recognition and processing tasks. Some of the common denoising techniques are filter methods, wavelet transform methods, statistical methods, DL methods, and so on.

Median filtering is a widely used technique for image denoising. The method works by choosing each pixel in an image and a small area around it, then arranging the pixels in that area by the size of their gray values, and then picking the middle value (i.e., the median) of the arranged area as the new value for that pixel.^40^ This operation is effective in removing the noise because the noisy pixel has a gray value that is very different from the normal values of the nearby pixels, whereas the median selected by median filtering is much more similar to the normal values of the nearby pixels.

#### 2.1.5 Resolution enhancement

Resolution enhancement is an image processing technique that aims to increase the sharpness and quality of an image to make it look more high‐resolution. Preserving the edge and shape information of an image is essential for correctly segmenting an image. The quality of its edges deteriorates after the image is denoised and its edge and shape information can be maintained by resolution enhancement. Discrete wavelet transform (DWT) is a frequently used method for resolution enhancement.

#### 2.1.6 MR bias field correction

“Magnetic field inhomogeneity” or “bias field” distortion in MRI data can cause more inconsistencies between images taken by the same scanner and the same patient.^41^ If the spatial frequencies of the actual image and the bias field are distinct, the bias field can be removed by filtering out spatial frequencies corresponding to the bias field, which is a technique called image bias field correction. Currently, common bias field correction techniques include N4 bias correction,^42^ joint elimination of MRI bias,^43^ model‐based correction methods, and DL methods.

In practical applications, Liang et al.^44^ proposed an unsupervised decomposition network for obtaining unbiased MRI images from biased data. The network consists of a segmentation part (predicting the probability of each pixel belonging to each category) and an estimation part (calculating the bias field), which are trained through alternating optimization. This method performs well in bias field estimation and correction. Mélanie Gaillochet^45^ explored modeling the bias field during the reconstruction process to reduce the sensitivity of the reconstruction to the bias field.

#### 2.1.7 Resolution adjustment of PET images

PET is an advanced technology used for functional and metabolic imaging, allowing the identification of biological targets. Typically, anatomical images exhibit higher resolution, while functional images have lower resolution. To maintain consistency in the size of multimodal images, researchers have proposed various adjustment methods.

For instance, Cai and colleagues employed an interpolation algorithm to enhance the resolution of PET images.^46^ Vivek Walimbe and his team introduced a resampling technique to decrease the resolution of CT images, aligning them with PET images.^47^ In contrast, David Mattes and associates combined these two approaches, enhancing the resolution of PET images while simultaneously reducing that of CT images. This achieved an optimal intermediate level for both image types.^48^ Additionally, applying bicubic spline interpolation to low‐resolution PET images can further improve their resolution.^49^

#### 2.1.8 Ultrasound image preprocessing

As the concept of fusion imaging technology was gradually integrated into clinical practice in the 1990s, ultrasonography was initially employed in the field of image fusion to identify different types of liver tumors.^50^ Ultrasound imaging offers real‐time performance, affordability, and avoids radiation exposure. In clinical practice, ultrasound‐guided procedures such as punctures and tumor resections are common.

However, the image quality of ultrasound is often suboptimal, making accurate lesion display and localization challenging. MRI provides high‐quality images with various modes, facilitating precise tumor localization. Nevertheless, MR‐guided surgery is costly and lacks real‐time capabilities. To address these limitations, researchers have proposed a novel approach that fuses preoperative MR/CT images with intraoperative or postoperative ultrasound images. This method not only ensures real‐time ultrasound guidance but also enhances accuracy by incorporating preoperative imaging data. In the research conducted by Andriy Fedorov and his team,^51^ preprocessing involved prostate segmentation on MRI images to obtain a smooth surface. Additionally, 3D ultrasound images of the prostate were acquired via rectal scanning, segmented, and further smoothed. The resulting prostate masks from both MRI and 3D ultrasound served as input for the registration algorithm, normalized to a consistent resolution.

### 2.2 Image fusion techniques

**FIGURE 1:**
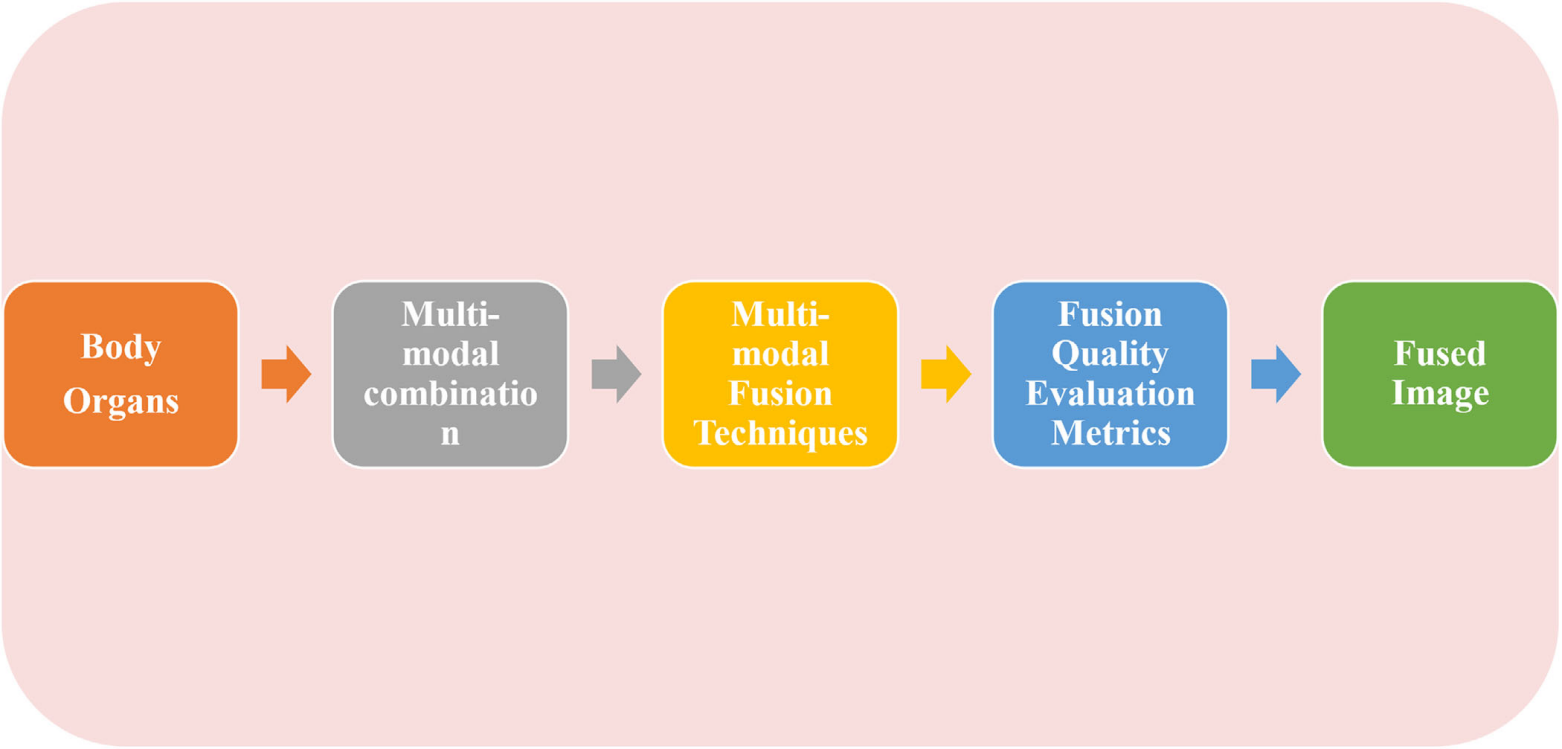
General multimodal medical fusion process.

Two criteria need to be met when performing MMIF: (1) the fused image should preserve all the relevant medical information from the input image; and (2) the fused image should not introduce any extra information that is not in the input image. The MMIF process and its different steps are explained below, and the whole MMIF process is illustrated in Figure 1.

The first step is to align the input source image by mapping it to a reference image to find and match corresponding images based on specific features used for the image fusion process.^52^ In the image decomposition step, the input image is split into sub‐images and fusion coefficients using a fusion algorithm, and then the fusion rules are applied to extract the important information and the variety of features of the sub‐images. In the fusion step, the fused image is reconstructed by combining the sub‐images using an inverse algorithm called image reconstruction.^53^

**FIGURE 2:**
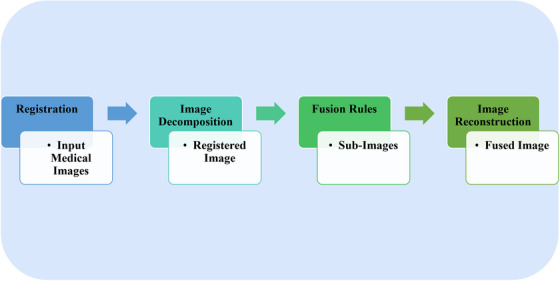
A sequential procedure for medical image fusion.

The image fusion method involves several basic stages that contribute to this aim. Figure 2 illustrates the main steps involved in the image fusion method.

Image fusion algorithms can be classified into three types based on the level of image fusion: pixel‐level, feature‐level, and decision‐level. Pixel‐level image fusion works on the image data at the pixel level of the image^54^ and is the basic type of image fusion. Feature‐level image fusion is the intermediate type of fusion, and it extracts the useful feature information from each image, such as edges, texture, etc., based on the specific imaging characteristics of each sensor.^55^ Decision‐level fusion is the highest type of fusion, and it further performs feature recognition, decision classification, and other processing on the target features of the source image, and then integrates the decision information of the individual source images for joint inference to obtain the inference result.

We have concentrated on six main methods of image fusion: frequency fusion, spatial fusion, decision‐level fusion, DL, hybrid fusion, and sparse representation fusion.

#### 2.2.1 Frequency‐based fusion methods

This method converts the input image to the frequency domain by computing the Fourier transform (FT) and then applies the fusion algorithm on the converted image and then gets the fused image by inverse FT.^56^ This method is divided into pyramid fusion methods and transform fusion methods. Pyramid fusion methods consist of Gaussian pyramid, Laplace pyramid, and hybrid pyramid depth synthesis,^57^ while transform fusion methods consist of wavelet decomposition, curvelet transform, and contourlet transform.^58^

#### 2.2.2 Spatial‐based fusion methods

Spatial‐based fusion methods work on the pixels of an image and process the pixel values to achieve the desired outcome.^59^ This method concentrates on processing features such as spatial distribution, structure, and texture in the image to obtain a better quality or more informative representation of the fused image. However, spatial domain fusion methods cause spatial distortion in the fused image, so current methods are aimed at reducing spatial distortion and obtaining a better fused image.

#### 2.2.3 Decision‐based fusion

Decision‐based fusion uses specific predefined criteria to evaluate each input image and then combines them into a globally optimal image based on the confidence level of each evaluation to produce a fused image. This technique employs certain rules defined before the fusion process to produce the maximum amount of information.^60^ Dictionary learning and Bayesian techniques are the most commonly used methods in decision‐based fusion. Dictionary learning is the automatic learning of a set of “dictionaries” or “atoms” from the data in order to represent the input data. Bayesian techniques, on the other hand, are statistical analysis methods based on Bayes' theorem, which are used in image fusion to optimize the fusion method, reduce uncertainty, and improve the outcomes. The core idea is to establish a relationship between the observed data and the prior information in order to infer the unknown or uncertain information.

#### 2.2.4 DL fusion methods

This method uses a multi‐layer architecture where each layer receives input from the layer below. DL enables the use of layered architectures and complex frame structures for large data processing.^61^, ^62^ DL fusion methods include CNNs, convolutional sparse representations (CSRs), and deep convolutional neural networks (DCCNs). Among these techniques, DL CNN models are favored for their trainability and good tunability.^63^

#### 2.2.5 Hybrid fusion methods

Because traditional multimodal image fusion methods do not produce satisfactory results, the basic idea of hybrid methods is to combine two or more fusion techniques to enhance the quality and performance of the fused images. The benefits of hybrid methods are to improve the visual quality and to reduce the artifacts and noise in the fused image.

#### 2.2.6 Sparse representation methods

The sparse representation (SR) method is to obtain an overcomplete dictionary from a sequence of images, thus achieving a stable and significant representation of the source image.^64^ The basic principle is to process the image signal as a linear combination of less significant atoms from a pre‐trained dictionary learning, where the sparse coefficients show the significant features of the input image.

Table 1 presents the pros and cons of each fusion method.

**TABLE 1:** The pros and cons of each fusion method.

Fusion method	Advantages	Disadvantages	Author (Year)
Frequency fusion method	Utilizes frequency domain information, provides global features; suitable for images of different resolutions.	May lose some local detailed information; relatively weak in modeling non‐linear relationships.	Princess et al. (2014)^56^
Spatial fusion method	Retains more local detailed information; strong in modeling spatial relationships.	Computational cost may be higher; challenges in issues like image registration.	El‐Gamal et al. (2016)^59^
Decision‐level fusion	Allows each modality to independently generate decisions; can address differences between modalities.	May not fully leverage inter‐modal correlated information; handling conflicts between modalities can be complex.	Bikash et al. (2019)^60^
Deep learning fusion method	Possesses powerful feature learning capability, able to extract high‐level abstract features from data; performs exceptionally well on large‐scale datasets.	Requires a large amount of annotated data for training; relatively lower model interpretability.	Zeiler et al. (2010)^63^
Hybrid fusion method	Combines multiple fusion strategies, overcoming limitations of a single method; exhibits strong flexibility.	May increase system complexity; adjusting parameters for each method can be cumbersome.	Yadav et al. (2020)^65^
Sparse representation method	Extracts sparse features from images, reducing dimensionality; used for feature selection and denoising.	Relatively weak in modeling complex relationships; requires some expertise in sparse representation selection and tuning.	Qiang et al. (2018)^64^

### 2.3 Application of multimodal technology

Although the research on multimodal imaging technology is multifaceted and multi‐disciplinary, multimodal conversion between CT and MRI is a promising field for the application of AI technology in medical imaging. The task involves mapping the original CT image to the target MRI image. However, cross‐channel image registration between MRI and CT presents challenges due to the significant variability in tissue and organ appearance resulting from different imaging mechanisms.

Yile Pan et al.^66^ addressed the challenge of collecting corresponding CT and MRI data in practical applications. To tackle the cross‐modal transformation of unpaired medical images, they proposed a cycle consistent generative adversarial network (CGN) that combines cyclic consistency generation. The main idea behind their unpaired multimodal image conversion method, known as “CSCGAN,” involves integrating the fine control characteristics of StyleGAN2 into the generator network of CycleGAN.^67^ They construct new generators and discriminators and incorporate a mixed attention mechanism. Notably, their approach achieves a dice similarity coefficient (DSC) of 0.74 ± 0.13, a Hausdorff distance (HD) of 10.31 ± 6.57, and a relative volume ratio (VR) of 0.17 ± 0.18.

To further leverage the interaction between different modal information for tumor detection, Chao Pan et al.^68^ proposed a multimodal detection framework called the Multi‐scale Intermediate Multimodal Fusion Detection Network (MIMFNet) based on the feature pyramid network (FPN).^69^ MIMFNet specifically focuses on detecting both benign (e.g., HCC) and malignant (e.g., hepatocellular adenoma, HCA) liver tumors. The architecture of MIMFNet comprises two main components: an intermediate multimodal fusion backbone and a multimodal enhanced feature pyramid network (EFPN). The fusion backbone consists of two parallel branches that fuse cross‐modal features from different stages between these branches. As the network propagates forward, it effectively maintains multimodal information, allowing different modalities to complement each other and enhance overall performance. Additionally, MIMFNet introduces multimodal global context coding and dual attention modules to further improve feature representation. The evaluation metric used by the authors is mean average precision (AP@0.5). When compared to other state‐of‐the‐art methods such as Cascade R‐CNN, Libra R‐CNN, and interlayer fusion approaches like HyperDenseNet^70^ and OctopusNet,^71^ MIMFNet achieves a higher AP.

While deep learning has made strides in medical image segmentation, these tasks demand substantial labeled data. However, due to constraints such as limited availability, time, and the efficiency of radiologists’ annotations, obtaining large, accurately annotated medical image datasets remains challenging. Consequently, the application of supervised learning‐based segmentation methods in routine clinical practice is restricted. In recent years, researchers have proposed innovative approaches to leverage multimodal information in medical images through contrastive semantic alignment. Notably, multi alignment encodes images into spatial feature maps and estimates local similarity between different modal images using multimodal contrastive learning, achieving effective multimodal alignment. Additionally, transferring annotations from one modality as pseudo labels to another enables unpaired multimodal medical image segmentation with a single set of annotations.

Jiajiao Zhang et al.^72^ introduced a novel multimodal contrastive self‐supervised method for medical image segmentation called Multimodal Comparative Domain Sharing (Multi ConDoS) generative adaptive networks. By integrating CycleGAN with the classic paired image translation model Pix2Pix, Multi ConDoS leverages the cyclic learning strategy of CycleGAN and the cross‐domain translation loss of Pix2Pix to enhance domain translation capabilities. Across various patterns in Hecktor and BraTS2018 datasets, Multi ConDoS consistently outperforms fully supervised, self‐supervised, and semi‐supervised baselines in terms of DSC and sensitivity (Sen).

Most existing research focuses on segmenting contrast‐enhanced images using publicly available datasets such as LiTS. However, many of these studies only consider the implicit relationships within a single imaging modality, without effectively utilizing the complementary features of multimodal MRI images. Xiao Xiaojiao^73^ proposed a multi‐task approach that combines features from multiple modalities in a network called Mt‐C‐Mmf. This model leverages multimodal contrast‐free MRI information for liver tumor segmentation and detection, addressing the challenges of ambiguity and specificity in tumor information from contrast‐free MRI images. Specifically, the DSC achieved by Mt‐C‐Mmf for segmentation is (81.98 ± 1.07)%, and the pixel‐wise accuracy (pAcc) is (93.72 ± 0.97)%. For tumor detection, the Intersection over Union (IoU) reaches (80.19 ± 1.46)%, and the classification accuracy is (90.36 ± 0.61)%. Additionally, the model demonstrates high sensitivity (90.83 ± 1.76)% and specificity (90.15 ± 0.87)%.

Accurate liver resection is an ideal treatment for liver cancer, and multimodal imaging technology helps to precisely locate tumors and detect all lesions as well.^74^, ^75^ Building upon previous research on liver 3D visualization, Fang Chihua et al.^76^ segmented, registered, and fused preoperative CTA (computed tomography angiography) information with MRI liver tumor data from patients. This fusion process resulted in a CT‐MRI integrated 3D model of the liver. The model seamlessly combines CT and MRI images in both time and space, intuitively and comprehensively displaying the size, location, and morphology of liver tumors. Additionally, it provides clear visualization of intrahepatic blood vessels, aiding in determining the spatial relationship between tumors and critical blood vessels within the liver. The model allows for repeated simulation and exploration of liver resection paths, enabling three‐dimensional and accurate dynamic analysis. Ultimately, it offers essential support for precise preoperative evaluation and the formulation of optimal surgical plans.

It is noteworthy that Aisha Siam et al.^77^ conducted a comprehensive scoping review of HCC multimodal machine‐assisted medical services studies spanning from January 2018 to August 15, 2023. After excluding non‐compliant studies, they analyzed a total of 10 studies, with eight originating from China and only two from Brazil. The research objectives primarily focused on disease prediction and disease classification or diagnosis. Additionally, these models were employed for predicting treatment response, determining survival rates, and staging the disease. CNNs emerged as the predominant deep learning architecture in these studies, with CT scans being the most frequently used imaging modality across six of the studies. Interestingly, while the studies did not report the combined use of multiple imaging methods, they demonstrated the capability to handle multiple biomarkers and clinical parameters.

## 3 SEGMENTATION METHOD BASED ON DEEP LEARNING

As AI and hardware advance, deep learning‐based medical image segmentation is attracting more and more research attention. Deep learning segmentation algorithms have strong feature extraction ability, which can fully exploit the image information and achieve precise and efficient medical image segmentation. Based on the development history of deep learning‐based medical image segmentation algorithms and the network architecture used, they are classified into CNNs, FCNs, DeepLab, U‐Net and its variants, and other segmentation methods.

### 3.1 CNN

For liver cancer image segmentation, CNN^78^ is not a specific segmentation method but a deep learning model for image processing and feature extraction, and most of the commonly‐used segmentation methods nowadays are based on the structure or improved versions of CNNs. This model, as one of the most representative neural networks, has made many breakthroughs in the field of image processing and analysis by introducing convolutional layers to reduce the training parameters. However, CNN‐based inputs depend on pixel blocks, which are much smaller than the whole image and cannot be regarded as a whole image, which limits the performance of pixel‐level classification and directly affects the segmentation results.

In the study by Lin et al.,^79^ a CNN based on the VGG16 framework was trained using a combination of 217 two‐photon excited fluorescence and second harmonic generation images. Remarkably, the classification accuracy for liver cancer differentiation exceeded 90%. This research demonstrates that the fusion of multiphoton microscopy and deep learning algorithms can effectively classify HCC differentiation levels, offering an innovative CAD approach. This research is distinctly innovative, yet its accuracy requires further enhancement.

Cole et al.^80^ chose GoogleNet as a pre‐trained model for the detection of multiple organs, including the liver, heart, and kidneys, in 3D CT volumes. To achieve this, they employed a strategy of segmenting each slice into small patches and labeling these patches. The features were then extracted from these patches using the pre‐trained model, and a support vector machine (SVM) model was trained and tested for classification. It is noteworthy that, when comparing different patch sizes, the method performed better with larger patch sizes. Yu‐Shiang Lin employed a binary classifier based on GoogLeNet (Inception‐V1) to categorize HCC histopathology images.^81^ The classifier achieved an accuracy of 91.37% (±2.49), sensitivity of 92.16% (±4.93), and specificity of 90.57% (±2.54) in HCC classification.

DCGAN (Deep Convolutional Generative Adversarial Network) is an unsupervised learning algorithm that combines deep CNNs with generative adversarial networks.^82^ The DCGAN generator produces synthetic images by convolving and upsampling random noise, and it is commonly employed in two‐dimensional image generation tasks. Building upon this foundation, Runnan He et al.^83^ proposed an enhanced network based on the 3D U‐Net architecture to address the issue of low segmentation accuracy in existing 3D U‐Net networks. In their approach, the network served as a discriminator within the generative adversarial network (GAN) framework, leading to the development of a semi‐supervised 3D liver segmentation optimization algorithm. Furthermore, to improve the quality of 3D abdominal synthetic images generated from random noise input, they designed a deep convolutional neural network (DCNN) based on a feature recovery method. The proposed algorithm underwent testing on the LiTS‐2017 and KiTS19 datasets, demonstrating a Dice score of 0.9424, surpassing other methods.

Researchers explored the use of the R‐CNN (region‐based CNN) model for liver cancer classification in a recent study published in June 2023.^84^ The R‐CNN demonstrated an impressive accuracy of 96.7% and exhibited reliable generalization capabilities. This means that it was able to classify liver cancer accurately almost all the time and should perform well on new, unseen data as well.

In a recent research, Maryam Fallahpoor et al.^85^ explored liver and liver lesion segmentation using deep learning on clinical 3D MRI data with the Isensee 2017 framework. At its core, the network employs a CNN and this model is grounded in a U‐Net‐based architecture. The study collected data from 128 patients, including T1w and T2w MRI images, and generated ground truth labels for liver and liver lesions. The deep learning model achieved an average DC of 88% for liver segmentation and 53% for liver tumors. Liver segmentation accuracy holds promise for dosimetry during radiation therapies and attenuation correction in PET/MRI scanners.

### 3.2 Fully convolutional neural network (FCN)

In 2015, Long^86^ proposed FCNs for image segmentation, which is regarded as a breakthrough in image segmentation technology. Its basic structure consists of input, convolution, pooling, and output. The main idea of FCN is to replace the fully connected layer at the end of the traditional CNN with the convolutional layer, and the whole network mainly comprises the convolutional layer and the pooling layer. The FCN can take any size of the input image, and produce an output image that matches the original image size. The skip connection structure within the network facilitates the restoration of the network's full spatial resolution. It integrates semantic information from deeper, more coarse layers with the appearance details from shallower, more granular layers, facilitating precise and detailed segmentation. This approach not only produces a label prediction for each pixel while preserving the spatial information of the original input image, but also subsequently restores the category of each pixel in the sampled feature image from abstract features, thereby extending classification from the image to the pixel level. Pre‐trained FCN models (e.g., using architectures like VGG, ResNet, or MobileNet) are available and can be fine‐tuned for specific tasks. FCN is also one of the most commonly used deep learning architectures, applied for liver and tumor segmentation from volumetric images.^87^, ^88^

In early practice, Avi Ben‐Cohen and his team investigated the performance of FCNs on a relatively small dataset.^89^, ^90^ The study compared FCNs to patch‐based CNNs and sparsity‐based classification schemes. The results showed a true positive rate of 0.86 and 0.6 false positives per case. Additionally, the average dice index was 0.89, the average sensitivity was 0.86, and the average positive predictive value reached 0.95. In 2020, Xin Dong et al.^91^ proposed a hybrid fully convolutional neural network (HFCNN) for liver tumor segmentation. This model can accept any input and produce accurate output, processing complete images instead of small blocks to avoid redundant estimation when blocks overlap. Additionally, it merges different scales by adding links to combine the last layer detection with finer measurement layers. The algorithm demonstrated highly accurate liver volume measurements, achieving 97.22%. Furthermore, the segmentation method exhibited an average DC of 0.92. The results highlight that the FCN performs exceptionally well with data variations, adjacent slices, and appropriate class weights. The accuracy achieved by using cascaded full convolutional neural networks (CFCNs) for liver tumor analysis, as proposed by Piyush Kumar Shukla,^92^ is 94.21%. The calculation time for analyzing each volume of liver tumor is less than 90 s. Additionally, the overall accuracy of the training and testing program on the 3DIRCAD dataset, which includes various volumes, is 93.85%. Recently, Hefu Xie et al.^93^ introduced a deep learning method that combines fully connected and CNNs for effective ALF prediction, achieving an accuracy of 94.8% and excellent generalization ability.

However, FCN also has some limitations: first, the network training is complex and the segmentation results are not precise enough, and it is not sensitive to the intrinsic details of the image; second, it does not consider the global context information, neglects the relationship between pixels, and lacks spatial consistency.

**FIGURE 3:**
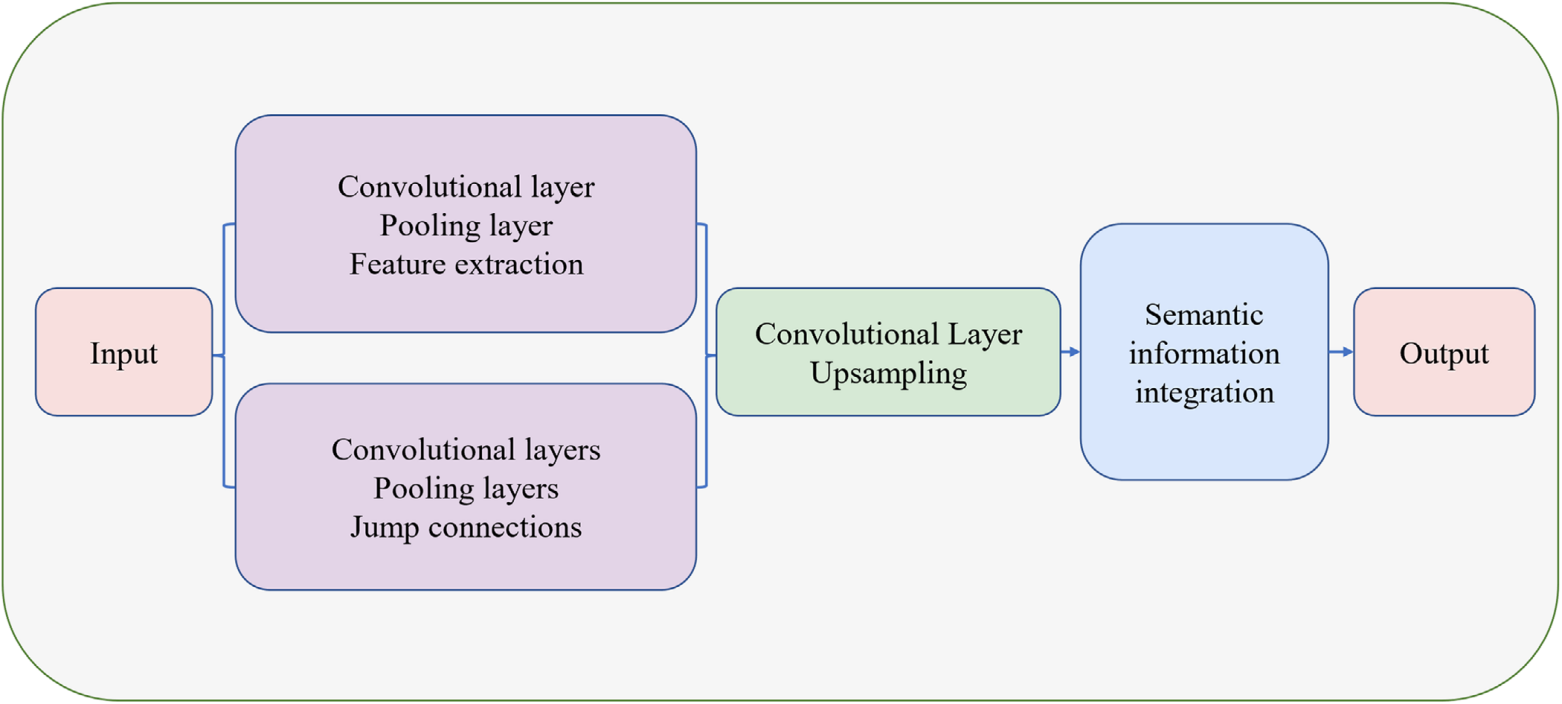
CNN and FCN.

Figure 3 briefly introduces the algorithms of CNN and FCN.

### 3.3 DeepLab series

Chen^94^ introduces DeepLab‐V1 network, which combines FCN with Conditional‐Random‐Field (CRF), and addresses the limitation of FCN's low segmentation accuracy by appending a fully‐connected CRF model after FCN. CRF refines the coarse segmentation maps from FCN, and produces more precise image segmentation results by building a CRF model at each pixel point in the map. The CRF refines the coarse segmentation map from FCN, and produces more precise image segmentation results by building a CRF model at each pixel point in the map.

DeepLab‐V1 applies the atrous convolution algorithm to increase the receptive field and obtain more contextual information of the image, and it can also avoid the problem of feature map resolution degradation caused by FCN during convolution and pooling. Moreover, due to the addition of atrous convolution in DeepLab‐V1, the running speed is greatly improved.

DeepLab‐V2 also uses the CRF model and the atrous convolution algorithm. Meanwhile, DeepLab‐V2 uses the Atrous‐Spatial‐Pyramid‐Pooling (ASPP) module, which obtains more spatial information by sampling the feature maps in parallel with different sampling rates of the atrous convolution and merging the output results. DeepLab‐V2 further enhances the segmentation effect by replacing the traditional network's VGG‐16 with a ResNet module.

DeepLab‐V3^95^ focuses on improving the use of atrous convolution in the model by proposing to gradually double the sampling rate of the cascade module, and at the same time expanding the ASPP module in the DeepLab‐V2 model, the performance of the model has been greatly improved, and thus it has been widely used in medical image segmentation tasks.

In practice, Wei Tang et al.^96^ proposed a method called the detection and segmentation laboratory (DSL), which combines Faster R‐CNN (Faster Region with CNN features) and DeepLab. The DSL approach involves two steps: first, to narrow down the liver segmentation area, Faster R‐CNN is employed to detect the liver region. Subsequently, the detection results are input into DeepLab for further segmentation. When evaluated on the MICCAISliver07 dataset, the DSL method achieved the following performance scores: VOE (Volume Overlap Error) of 80.2%, RVD (relative volume difference) of 94.1%, ASD (average surface distance) of 80.5%, RMSD (root mean square distance) of 76.4%, and MSD (maximum surface distance) of 71.9%. Notably, these scores were significantly better than those obtained by DeepLab.

Thanks to global information modeling derived from self‐attention mechanisms (SAMs), Transformers have recently achieved significant results in natural language processing and computer vision. Reza Azad et al.^97^ proposed a novel pure Transformer called TransDeepLab, which is similar to DeepLab for medical image segmentation. The authors extended DeepLabv3 by incorporating a layered Swin Transformer with a shift window and modeling the ASPP module. Notably, TransDeepLab demonstrates excellent performance in multiple organ segmentation using the Synapse dataset, achieving a DSC of 80.16 and a HD of only 21.25. Moreover, this approach significantly reduces model complexity.

### 3.4 U‐net series

Ronneberger introduced the U‐Net network in 2015.^98^ It is a modified and expanded version of the FCN network and, like FCN, U‐Net does not have a fully‐connected layer; its main components are the convolutional layer and the pooling layer, and its network structure consists of encoders and decoders. The network mainly consists of the left encoder part and the middle skip connection. The encoder extracts features from the input image layer by layer, and the decoder restores the image information layer by layer. The middle skip connection merges the bottom layer information in the encoder structure with the higher layer information in the decoder structure to improve the segmentation accuracy. U‐Net network follows the FCN idea, but by adding more channels it allows the context information of the network to flow to higher resolution layers, and by replacing the pooling operation with the upsampling operation it learns better and requires only a small amount of labeled data.

Zhou^99^ improved U‐net and proposed U‐Net++ in 2018. The improvement is the skip connection part in the middle of the U‐net network structure. U‐Net++ uses a kind of densely connected skip connection, which overlays features from different layers, so that the encoder and decoder sub‐networks are more connected, and the semantic gap between the feature mapping of the encoder and decoder sub‐networks is reduced.

Huang^100^ proposed U‐Net3+ in 2020. U‐Net3+ uses full‐scale skip connectivity and deep supervision to address these problems. Full‐scale skip connectivity combines low‐level details from features at different scales with high‐level semantics; deep supervision learns feature representations from fully aggregated feature mappings. The network parameters are decreased and the computational efficiency is increased, and the segmentation results are also improved well.

Çiçek^101^ proposed 3DU‐Net, 3DU‐net is an extension of the classic U‐net framework which enables 3D volume segmentation. All 2D operations are replaced with corresponding 3D operations, that is, 3D convolution, 3Dmaxpooling, and so forth, to obtain a three‐dimensional segmented image.

U‐Net++ and U‐Net 3+ have been actively applied in liver and liver segmentation. Li et al.^102^ utilized the U‐Net++ architecture with an attention‐aware mechanism for liver segmentation from CT images. Similarly, Huang et al.^103^ employed the U‐Net 3+ architecture for liver segmentation, achieving state‐of‐the‐art performance compared to other U‐Net architectures.

Regarding applications, Li et al.^104^ proposed a U‐Net variant model called the H‐DenseUNet for the segmentation of liver and liver tumor CT images. In this model, image feature extraction is performed using DenseNet‐169. The H‐DenseUNet consists of two parts: a 2D DenseUNet model, which efficiently extracts feature information from images, and a 3D DenseUNet model, which aggregates contextual information across polymer elements. By effectively extracting 3D feature information from the liver and liver tumor regions, the H‐DenseUNet achieves accurate segmentation.

Liu et al.^105^ proposed an algorithm called GIU Net, which combines the 2DU Net deep learning model with traditional image segmentation techniques such as graph cutting, for liver segmentation. First, the U‐Net model is used to obtain the probability distribution map of the liver region. Then, based on the probability distribution map and contextual information from the liver image, the energy function required for the graph cutting algorithm is created. Finally, the liver region is segmented by optimizing the energy function.

In response to the limitations of traditional segmentation methods – such as the threshold method, region growth, and K‐means – which require manual parameter tuning for segmenting liver and liver tumors with complex image features, Fan Tongle proposed innovative solutions.^106^ These traditional methods rely solely on pixel grayscale information, leading to ineffective segmentation. Additionally, conventional U‐Net models do not fully consider multi‐scale image information, resulting in semantic gap issues and suboptimal segmentation accuracy.

Fan Tongle introduced an embedded liver segmentation model based on multi‐scale semantic information – the MSN‐Net model. When tested on the LIST dataset, the MSN‐Net achieved a DC of 0.9424 and an IOU of 0.9075. Remarkably, when compared to SegNet, U‐Net, Res Unet, U‐Net++, and DenseNet‐103 under the same conditions, this model not only achieves the highest segmentation accuracy but also ensures a low number of parameters.

We have observed that attention modules are a prominent topic in U‐Net research. Kasun Hettihewa et al.^107^ proposed a novel network based on U‐Net, called the Multi Attention Network (MANet). MANet combines attention mechanisms to emphasize important features while suppressing irrelevant ones, specifically for tumor segmentation tasks. However, the authors noted a significant performance gap between slice‐based segmentation and volume‐based segmentation. This challenge arises due to the high variability in data sources, including variations in the shape of liver tumors and intensity levels. Consequently, addressing these issues is crucial for generalizing the model's performance in real clinical environments.

For segmentation tasks, however, there have been few significant breakthroughs in network structure since the release of UNet. The more structural modifications we introduce, the greater the risk of overfitting. Consequently, a group of researchers increasingly believes that the key to improvement lies in understanding the data and adopting appropriate preprocessing and training methods for medical data. Fabian Isensee et al.^108^ proposed a robust adaptive framework called nnUNet, which builds upon the 2D UNet and 3D UNet architectures. Although not the most recent research, this framework performs admirably. While it doesn't introduce a novel network structure, it leverages specific techniques to unify segmentation tasks and achieve excellent results across various applications. The widespread adoption of nnUNet's code and its proven effectiveness prompt us to reflect deeply. In reality, the purported innovations in network structure over the years often boil down to overfitting – a pitfall that paper authors sometimes fall into.

### 3.5 Transformer

One of the main limitations of CNNs is that they have a restricted receptive field to learn distant spatial correlations in images. A common way to overcome this problem is to use a null convolution instead of a standard convolution, but this leads to information discontinuity and grid effects. Recently, some researchers have borrowed the idea of Transformer from natural language processing, which can capture global contextual information effectively, and combined it with CNN to achieve a balance of local and global features.

TransUNet^109^ is a network architecture that integrates the benefits of Transformer and U‐Net. It keeps the U‐Net encoder‐decoder structure and adds a Transformer module to the last layer of the encoder to recover the global information that is lost and to perform accurate image segmentation.

TranBTS^110^ is a successful fusion of 3DCNN and Transformer for the multimodal brain tumor segmentation problem in MRI, which not only leverages the strength of 3DCNN in modeling local context, but also uses Transformer to learn global semantic relationships.

Swin‐UNet^111^ is the first pure Transformer that consists of a U‐shaped encoder‐decoder structure with skip connections, similar to the U‐Net architecture, which uses the encoder structure to extract contextual features and the decoder to restore the feature map dimensions. Swin‐UNet's U‐shaped structure merges the high‐level and low‐level features while avoiding the drawbacks of convolution, and has shown better performance than other Transformer methods on several medical image segmentation tasks.

The parallelized training of Transformers better captures comprehensive information. This architecture, entirely based on attention mechanisms, has the ability to suppress irrelevant background while highlighting useful features. This proves beneficial in rapidly and accurately locating tumors in liver tumor segmentation tasks.^112^

TransLiver^113^ is a hybrid transformer model for multi‐phase liver lesion classification. This study proposes TransLiver, a model designed to classify focal liver lesions (FLLs) using CT scans. FLLs can be either benign or malignant, and accurate early diagnosis is crucial. The TransLiver model integrates a modified Transformer backbone with complementary convolutional modules. It connects transformer blocks with convolutional encoders and down‐samplers. For multi‐phase fusion, TransLiver uses cross‐phase tokens to enhance communication between different imaging phases. The model achieved an overall accuracy of 90.9% on an in‐house dataset comprising four CT phases and seven liver lesion classes.

In recent research, a Transformer‐based deep learning approach has been applied to predict post‐liver transplant risk factors.^114^ The study aimed to predict multiple risk factors after a liver transplant, and the authors proposed a novel multi‐task TabTransformer deep learning model for predicting five risk factors post liver transplant. The proposed model achieved high accuracy and maintained a good balance in predicting all five post‐transplant risk factors, with a maximum accuracy discrepancy of only 2.7%. Another recent study introduces the SDR‐Former framework,^115^ specifically designed for liver lesion classification in 3D multi‐phase CT and MR imaging with varying phase counts. The SDR‐Former, proposed by the author, utilizes a streamlined Siamese neural network (SNN) to process multiphase imaging inputs and introduces a new adaptive phase selection module (APSM). The ability of this model to adapt to a range of phase conditions is a significant advantage.

**FIGURE 4:**
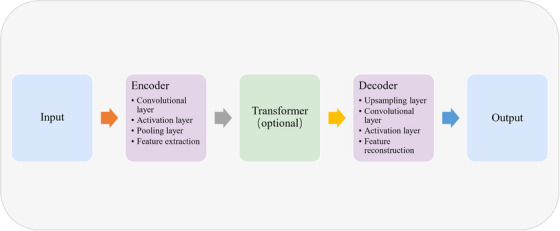
U‐Net and its improved versions.

The above content can be briefly summarized as shown in Figure 4.

### 3.6 GAN

GAN^116^ consists of two sub‐networks, the generator and the discriminator, which work in an adversarial manner. The generator produces images and feeds them to the discriminator, which decides whether the input images are real or generated, and both are optimized based on the adversarial process. Yang^117^ trained two separate networks: a generator network and a discriminator network with U‐Net as the backbone, by merging multimodal images as multi‐channel inputs. The generator network is responsible for predicting the segmentation results, while the discriminator network will determine whether the segmentation mask is the model prediction or the ground truth. Huo^118^ used Patch‐GAN as another discriminator to guide the training process of the network. The GAN‐based approach leads to more reliable segmentation due to the extra constraints on the discriminators, but training the additional discriminators requires more memory. In the previous text, we have introduced the DCGAN.^82^ GANs architecture has been proved to achieve good performances in the liver and liver tumor segmentation.^87^, ^102^, ^119^

In the study by Ugur Demir et al.,^120^ they proposed a new segmentation method that utilizes a SAM combining Transformer with GANs. This approach allows the network to aggregate high‐dimensional features and provide global information modeling. Their model achieved a high DC of 0.9433, a recall rate of 0.9515, and an accuracy of 0.9376, outperforming other Transformer‐based methods. And the proposed hybrid architecture (i.e., the combination of GAN and transformer) can potentially be applied to various medical image segmentation tasks outside of liver CT.

### 3.7 Segment anything model (SAM)

Due to the high expense and time required to collect and manage medical images, there is a growing interest in research on “zero‐shot”, that is, zero‐sample, fundamental image segmentation models.^121^ In April 2023, Meta launched the SAM, a new fundamental model for AI that achieves remarkable segmentation results on various natural image datasets when trained on a massive dataset. The SAM model learns to train following the inspiring idea of prompt from Chat‐GPT, which stands for prompt‐based learning, that is, cue‐based learning, which differs from traditional supervised learning by transforming the downstream tasks into the form of pre‐trained tasks, constructing the data from the downstream tasks into the form of natural language with specific templates, and fully utilizing the ability of the pre‐trained model itself, and thus being adopted by the GPT‐3 team. Prompt not only reduces the sample size of model training, and allows for training with fewer samples or even zero samples, but also enhances the model's generalizability. SAM leverages this cutting‐edge technology route to achieve a breakthrough in the fundamental CV technology, and has wide generalization and zero‐sample transfer capabilities. A notable limitation of SAM is that it can only handle 2D images, while many medical images are 3D, and although the algorithm can be extended to 3D in several ways, the possible extension poses many significant challenges to algorithm development.

Medical images are difficult to segment due to diverse imaging modalities, delicate anatomical structures, unclear and complex boundaries, and wide range of object scales. To assess the performance of SAM for medical image segmentation, several universities, including Shenzhen University, have jointly created one of the largest medical image segmentation datasets to date, COSMOS 553K, and based on this dataset, we have conducted a comprehensive, multi‐faceted, and large‐scale detailed evaluation of SAM.^121^ The evaluation results show that, although SAM has the potential to become a generalized medical image segmentation model, its performance in medical image segmentation tasks is currently unstable, especially the fully automatic Everything segmentation mode is not suitable for most medical image segmentation tasks, in which SAM has poor recognition of medical segmentation targets. Therefore, the research of SAM in medical image segmentation should focus on how to effectively use a small number of medical images to fine‐tune the SAM to improve the reliability of the model, and build a SAM model belonging to medical images.

MedSAM is a study of applying SAM to medical image segmentation.^121^ The study proposes a simple fine‐tuning method to adapt SAM to generalized medical image segmentation tasks and conducts extensive experiments on 21 3D segmentation tasks and nine 2D segmentation tasks, with DSCs of the two of them being, respectively, 22.5% and 17.6%, which are better than the default SAM model, proving that MedSAM segmentation is better than the default SAM model.

**FIGURE 5:**
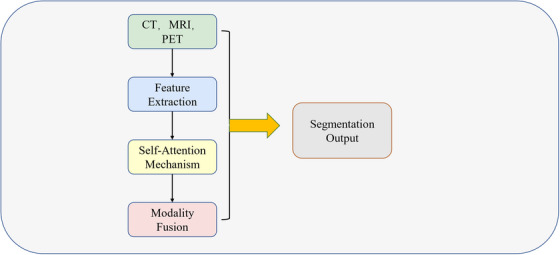
Segment anything model (SAM).

In the field of liver cancer multimodal image segmentation, SAM plays a crucial role. SAM can be applied for modality fusion, especially in handling images from different modalities. Its SAM helps capture local and global correlations in the image, improving segmentation accuracy. Additionally, SAM aids the model in understanding long‐range dependencies, particularly beneficial for large lesions or across multiple slices. SAM effectively models spatial relationships between different regions in the image, enhancing segmentation performance and interpretability. However, the application of SAM may face challenges in computational requirements and hyperparameter tuning, requiring in‐depth experimentation and adjustments to adapt to the specific characteristics of liver cancer image segmentation tasks. SAM mainly consists of the following steps, as shown in Figure 5.

Table 2 summarizes the principles of operation and advantages/disadvantages of each segmentation technique.

**TABLE 2:** The principles of operation and advantages/disadvantages of each segmentation technique.

Method	Principle of operation	Advantages	Disadvantages	Author (year)
Convolutional neural network (CNN)	Uses convolutional layers for feature extraction and image processing, but has limitations in handling the overall image.	Powerful ability to learn local features, relatively fast training process, particularly suitable for smaller images.	Relatively limited in capturing global information, may perform poorly in medical image segmentation tasks that require context information.	Szegedy et al. (2014)^78^
Fully convolutional neural network (FCN)	Introduces convolutional layers to replace fully connected layers, enhances spatial resolution through skip connections, achieving pixel‐level classification.	Capable of handling variable‐sized input images, achieving pixel‐level classification.	Training can be challenging, limited segmentation result accuracy, lacks spatial consistency.	Long et al. (2015)^86^
DeepLab series	Combines FCN with conditional random fields, utilizes dilated convolutions, and hole algorithm to enhance receptive field and accuracy.	Improves segmentation accuracy by introducing global contextual information.	Training can be relatively challenging, and there is still room for improvement in accuracy.	Chen et al. (2016)^94^
U‐Net series	Combines encoder and decoder, merges low‐level and high‐level information through skip connections, suitable for scenarios with limited annotated data.	Suitable for scenarios with limited annotated data, exhibits good learning performance.	Performance may be unstable under fully automatic segmentation mode.	Zhang et al. (2021)^98^
Transformer	Combines CNN and transformer, integrates local and global features by introducing transformer modules.	Enhances the representation capability of global contextual information.	Requires further improvements in medical image segmentation, such as adapting to 3D images.	Chen J et al. (2021)^109^
Generative adversarial network (GAN)	Includes a generator and a discriminator, enhances segmentation performance through adversarial training, supports multi‐channel input and additional discriminators.	Improves segmentation performance and enhances segmentation robustness.	Training additional discriminators requires more memory, and the receptive field is limited.	Li et al. (2022)^116^
Segment‐all models (SAM)	Based on prompt‐based learning, utilizes a foundational model through zero‐shot learning, assembles downstream tasks into pretraining tasks in natural language form through prompts.	Demonstrates excellent performance in zero‐shot learning, reduces the sample size, and enhances generalizability.	Performance is unstable in medical image segmentation, and perceptual capability is relatively poor under fully automatic segmentation mode.	Mazurowski et al. (2023)^121^

## 4 EVALUATION METRICS AND DATASETS

In this section, we explain the relevant metrics that we used to assess the methods of the chosen articles. Some of the metrics that we discuss here are based on the four basic components of the confusion matrix, namely, true positives (TP), true negatives (TN), false positives (FP), and false negatives (FN).

### 4.1 Sensitivity (SN)

Sensitivity, also known as the true positive rate (TPR), is the proportion of true positive predictions to the total number of positives.

$$SN=\frac{TP}{TP+FN}=\frac{TP}{P}$$

### 4.2 Specificity (SP)

Specificity, or the true negative rate (TNR), is the proportion of correct negative predictions to the total number of true negatives.

$$SP=\frac{TN}{TN+FP}=\frac{TN}{N}$$

### 4.3 Volumetric overlap error

VOE is calculated as a percentage and is given by the following equation:

$$VOE=\left(\left(\frac{\left|A\cap B\right|}{\left|A\cup B\right|}\right)-1\right)\times 100$$

where A represents a pixel in the segmented region and B a pixel in the real region. According to the described equation, the VOE calculates the intersection of the pixels in the segmented region (A) with those in the real region (B) relative to the union of the pixels in both the segmented (A) and real regions (B). A VOE value of zero indicates perfect segmentation, whereas a value of 100 signifies complete absence of segmentation.

### 4.4 DSC

The DSC is used to calculate the overall performance score of the model.

$$DSC=\frac{2\left|A\cap B\right|}{\left(\left|A\right|+\left|B\right|\right)}$$

A represents the segmented pixel and B the true pixel. The equation demonstrates that the DSC is calculated by dividing the intersection of these pixels by the total number of pixels in both the segmented and true regions. A Dice similarity score of “0” indicates poor segmentation, while a score of “1” signifies perfect segmentation.^122^

### 4.5 Jaccard similarity coefficient

The Jaccard coefficient, also known as “Intersection over Union (IoU),” is a metric used to evaluate the similarity between the segmentation result and the ground truth region by measuring the proportion of common elements to their union.

$$J\left(A,B\right)=\frac{\left|A\cap B\right|}{\left|A\cup B\right|}=\frac{\left|A\cap B\right|}{\left|A\right|+\left|B\right|-\left|A\cap B\right|}$$

### 4.6 Average symmetric surface distance

This metric is derived from the surface voxels of the segmented image A, with surface voxel S(A), and the surface voxels of the ground truth image B, with surface voxel S(B).

$$\begin{array}{ccc} & & ASD\left(A,B\right)=\frac{1}{\left(\left|S\left(A\right)\right|+\left|S\left(B\right)\right|\right)}\\ & & \;\times \left(\sum_{S_{A}\in S\left(A\right)}d\left(S_{A},S\left(B\right)\right)+\sum_{S_{B\in }S\left(B\right)}d\left(S_{B},S\left(A\right)\right)\right) \end{array}$$

where $d(s_{B},S\left(A\right))$ is the Euclidean distance as shown in the following equation:

$$d\left(v,S\left(A\right)\right)=\min_{S_{A}\in S\left(A\right)}\left|\left|v-S_{A}\right|\right|$$

For the set of surface voxels S(A) of image A, the same distance is computed for the set of surface voxels S(B) of image B to achieve symmetry. The Average Symmetric Surface Distance (ASD) is then defined as the mean of all these computed distances. An ASD value of “0” indicates perfect segmentation. ASD is measured in millimeters.

### 4.7 Root mean square symmetric surface distance

The root mean square (RMS) of the symmetric surface distance is also calculated in millimeters and is defined as

$$\begin{array}{ccc} & & RMS\left(A,B\right)=\sqrt{\frac{1}{\left(\left|S\left(A\right)\right|\right)+\left|S\left(B\right)\right|}}\\ & & \;\times \sqrt{\sum_{S_{A}S\left(A\right)}d^{2}\left(S_{A},S\left(B\right)\right)+\sum_{S_{B}\in S\left(B\right)}d^{2}\left(S_{B},S\left(A\right)\right)} \end{array}$$

From the above equation, it can be concluded that the root‐mean‐square distance is calculated in the same way as the mean surface distance, except that the Euclidean distance is squared. The root of the mean symmetric distance gives the root mean square symmetric surface distance. If the value of the root mean square symmetric surface distance is “0”, it means that there is a perfect segmentation.

### 4.8 Maximum symmetric surface distance

The maximum plane of symmetry distance, also known as the HD,^123^ is given by

$$\begin{array}{ccc} MSSD\left(A,B\right) & = & max\left\{\max_{S_{A}\in S\left(A\right)}d\left(S_{A},S\left(B\right)\right)\right.\\ & & +\left.\max_{S_{B}\in S\left(B\right)}d\left(S_{B},S\left(A\right)\right)\right\} \end{array}$$

The distance between the surface voxels S(A) and S(B) of the segmented image A and the ground truth image B is computed using the Euclidean distance, with the largest of these distances selected. This measurement is crucial in surgical planning applications as it helps to determine the worst‐case error.

### 4.9 Area under curve

A receiver operating characteristic (ROC) curve is a graphical representation illustrating the trade‐off between true positive and false positive rates of a classifier. For probabilistic classifiers, the ROC curve displays various points at different thresholds. The area under the ROC curve (AUC) is a scalar value summarizing the performance of such classifiers. It is calculated by integrating the ROC curve using the trapezoidal method.^124^

### 4.10 Datasets

Available liver datasets generally offer a limited number of images and reference segmentations, or provide no reference segmentations, compared to datasets for other organs. Table 3 lists some commonly used liver datasets.^125^

**TABLE 3:** Some commonly used liver datasets.

Dataset name	Description
LiTS (liver tumor segmentation)	Dataset composed of CT scan images of the liver and liver tumors, used for segmentation tasks.
MICCAI 2017 LiTS challenge dataset	Dataset related to the LiTS dataset, associated with the MICCAI 2017 Challenge, aimed at advancing research in liver tumor segmentation.
3Dircadb	3D CT scan images containing liver lesions, used for research in medical image processing and segmentation.
SLIVER07	Dataset composed of CT scan images of the liver and liver tumors, used for segmentation tasks.
TCIA CTS‐LITS Dataset	Dataset containing CT scan images with liver lesions, suitable for liver segmentation tasks.

## 5 LIMITATIONS AND FUTURE PERSPECTIVES

Deep learning's use in image segmentation for liver and HCC has seen significant growth recently. However, several issues need resolution before its integration into clinical practice. A common limitation in this field is the retrospective design of most studies, which may introduce selection bias in patient populations.^126^ Health datasets often face bias and quality issues, increasing the risk of model overfitting.^127^ Future studies would benefit from larger patient samples collected through prospective multicenter trials to enhance reliability.^128^ ML and deep learning rely on a foundation of extensive data. However, the majority of current research is conducted on small, single‐center datasets. Establishing high‐quality datasets at regional or even international levels is crucial for achieving significant breakthroughs at the data level. The creation of high‐quality public databases could also help, though these datasets require quality checks for validity and representativeness.^129^, ^130^ Additionally, privacy and ethical concerns must be addressed.^131^

Another challenge is the models' lack of interpretability. Described as “black boxes,” these models often obscure the decision‐making process based on inputs. Explainability is vital for building trust among physicians who may rely on these computer‐assisted diagnostic systems. Despite the superior performance of deep learning in image analysis, it remains a “black box” at present. The numerous parameters involved make it difficult for humans to understand the details of how deep learning analyzes data and makes decisions. Therefore, the precondition for broad acceptance by clinical professionals and patients is the “whitening” of deep learning.^132^ Furthermore, the implementation of AI‐based tools in clinical settings raises questions about liability, especially when discrepancies arise between physician assessments and tool recommendations, potentially leading to legal complications.^133^

In real clinical scenarios, it is essential to perform whole (i.e., 3D) liver segmentation. Using 2D segmentation can lead to boundary indentations and inaccuracies in the final results. When applying AI for liver segmentation in clinical practice, the focus should be on 3D segmentation, and any obstacles should be addressed. The prevalence of 2D datasets is primarily due to the scarcity of prepared 3D datasets available in online libraries, especially for MRI images.

Despite these challenges, deep learning holds great potential for liver and HCC image segmentation, as our review indicates. It can advance precision and personalized medicine, supporting clinical practice and optimizing costs and resources.^134^ Integrating models with clinicopathological data and established clinical scores or biomarkers shows promise.

Thematic analysis of PubMed search results using the latent Dirichlet allocation method revealed a decreasing trend in segmentation as a topic in AI‐related liver imaging research since the late 2010s. This shift, influenced by improvements in automated segmentation techniques using deep learning, suggests a transition from technology development to application, likely to yield greater clinical relevance.^135^

## 6 CONCLUSION

This paper offers an extensive overview and analysis of deep learning in multi‐modal fusion image segmentation for liver cancer. By reviewing numerous literatures, we conclude:

1. Deep learning demonstrates significant potential in liver cancer image segmentation. Techniques like CNN, FCN, DeepLab, U‐net, Transformer, GAN, SAM have enabled accurate segmentation from multimodal images, including CT, MRI, and PET. These developments enhance liver cancer diagnosis and treatment precision, potentially improving patient survival rates and quality of life.
2. Multi‐modal fusion offers considerable advantages in liver cancer image segmentation. Combining information from various image modalities not only heightens segmentation accuracy but also enriches tumor tissue descriptions. Fusion methods like feature‐level and decision‐level fusion add versatility to multimodal image segmentation.

However, challenges such as limited data, inaccurate labels, generalization performance, and model interpretability persist. Additionally, privacy and ethical considerations in patient data usage are crucial.

Future research should focus on overcoming these practical application challenges. Interdisciplinary collaboration, merging medicine, and computer science, will be pivotal in advancing liver cancer diagnosis and treatment.

In summary, deep learning in multimodal fusion image segmentation for liver cancer holds promising prospects. Though challenges remain, continued research and innovation are expected to overcome these hurdles, enhancing medical services for liver cancer patients and furthering medical image processing.

## AUTHOR CONTRIBUTIONS

**Chaopeng Wu**: Conceptualization; methodology; investigation; data curation; writing—original draft; visualization. **Qiyao Chen**: Conceptualization; methodology; investigation; data curation; writing—original draft. **Haoyu Wang**: Conceptualization; investigation. **Yu Guan**: Investigation. **Zhangyang Mian**: Investigation. **Cong Huang**: Investigation. **Changli Ruan**: Writing—review and editing. **Qibin Song**: Writing—review and editing. **Hao Jiang**: Writing—review and editing; project administration; funding acquisition. **Jinghui Pan**: Conceptualization; writing—review and editing; project administration. **Xiangpan Li**: Writing—review and editing; project administration; funding acquisition. All authors have read and consented to the published version of the manuscript.

## CONFLICT OF INTEREST STATEMENT

The authors declare no conflicts of interest.

## ETHICS STATEMENT

This study adhered to the ethical guidelines set forth by the Medical Ethics Committee of Renmin Hospital of Wuhan University. This study adhered to the ethical guidelines set forth by the Medical Ethics Committee of REDACTED.

## Data Availability

Research data are currently unavailable.
